# Patient-derived olfactory mucosa for study of the non-neuronal contribution to amyotrophic lateral sclerosis pathology

**DOI:** 10.1111/jcmm.12488

**Published:** 2015-03-25

**Authors:** Vega García-Escudero, María Rosales, José Luis Muñoz, Esteban Scola, Javier Medina, Hena Khalique, Guillermo Garaulet, Antonio Rodriguez, Filip Lim

**Affiliations:** a Departamento de Biología Molecular, Universidad Autónoma de MadridMadrid, Spain; b Centro de Biología Molecular “Severo Ochoa” (C.S.I.C.- U.A.M.), Universidad Autónoma de MadridMadrid, Spain; c Departamento de Neurología, Hospital General Universitario Gregorio MarañónMadrid, Spain; d Departamento de Otorrinolaringología, Hospital General Universitario Gregorio MarañónMadrid, Spain

**Keywords:** olfactory mucosa, amyotrophic lateral sclerosis, non-cell autonomous toxicity, SOD-1 neurotoxicity, inflammation-responsive promoter

## Abstract

Amyotrophic lateral sclerosis (ALS) is a degenerative motor neuron disease which currently has no cure. Research using rodent ALS models transgenic for mutant superoxide dismutase 1 (SOD1) has implicated that glial–neuronal interactions play a major role in the destruction of motor neurons, but the generality of this mechanism is not clear as SOD1 mutations only account for less than 2% of all ALS cases. Recently, this hypothesis was backed up by observation of similar effects using astrocytes derived from post-mortem spinal cord tissue of ALS patients which did not carry SOD1 mutations. However, such necropsy samples may not be easy to obtain and may not always yield viable cell cultures. Here, we have analysed olfactory mucosa (OM) cells, which can be easily isolated from living ALS patients. Disease-specific changes observed when ALS OM cells were co-cultured with human spinal cord neurons included decreased neuronal viability, aberrant neuronal morphology and altered glial inflammatory responses. Our results show the potential of OM cells as new cell models for ALS.

## Introduction

Amyotrophic lateral sclerosis (ALS), or Lou Gehrig's disease, is a neurodegenerative disorder of unknown origin characterized by progressive degeneration of upper motor neurons in the motor cortex and lower motor neurons in the brainstem and the spinal cord, initiating in mid-age life 1. It results in muscle paralysis and ultimately death because of respiratory failure, most commonly within 3–5 years of diagnosis 2.

The classical division of ALS into familial or sporadic types has been made depending on patient family history. Approximately, 90% of ALS patients are considered sporadic (sALS) as they appear to occur randomly throughout the community, whereas the remaining 10% are familial (fALS) cases 3, showing autosomal dominant inheritance in the majority of instances. Of these, ∽12% are associated with mutations in the Cu/Zn superoxide dismutase (SOD-1) gene 4, which seem to confer toxic gain of an unknown function rather than loss of normal SOD1 activity 5. Other genes, including TARDBP (TAR DNA-binding protein 43) 6, FUS (fused in sarcoma) 7,8 and angiogenin (ANG) 9 have also been implicated in fALS. Recently, a large GGGGCC repeat expansion in the first intron of the C9orf72 gene has been reported to be the most common genetic cause of ALS, accounting for approximately 40% of fALS and 7% of sALS 10–12. Sporadic and familial ALS cases are however, clinically indistinguishable, suggesting similar underlying pathophysiological mechanisms of neurodegeneration.

While several pathogenic processes for the motor neuron degeneration observed in ALS have been proposed, one of the most prevailing hypotheses supports a non-cell autonomous process: neighbouring astrocytes are thought to play a major role in disease progression 13–18. Nevertheless, the basis for the specificity of astrocytes and other glial cells in the selective degeneration of motor neurons remains unclear. Indeed, the molecular mechanisms underlying neuronal death in ALS are presently not fully determined and appear to be multifactorial. Glutamate excitotoxicity, mitochondrial dysfunction 19, accumulation of intracellular protein aggregates 20, oxidative stress 21, hypoxia 22,23, alterations in RNA metabolism 24, impaired axonal transport 25, growth factor deficiency 26 and neuroinflammation 27 have all been proposed to be involved.

Evidence of inflammation is observed in post-mortem tissue 28–31, in cerebrospinal fluid 32,33 and in blood samples 34 from fALS and sALS patients. These observations are in agreement with previous observations of neuroinflammation in rodent models of ALS 35,36. In transgenic mice expressing mutant SOD-1, increased levels of toll-like receptors (TLRs) are detected 37. Microglial neurotoxic inflammatory responses have been suggested to be facilitated *via* TLR2 38. In addition, it has been shown that mutant SOD-1 binds to CD14, which is a co-receptor of TLR2 and TLR4, and that the microglial activation mediated by mutant SOD-1 can be attenuated using TLR2, TLR4 and CD14 blocking antibodies 39. In accordance, Casula *et al*. have reported an up-regulation of TLR2 and TLR4 as well as other pro-inflammatory molecules such as Receptor for Advanced Glycation End products and High Mobility Group protein B1 in reactive glia in the spinal cord of sALS victims 30. One unanswered question, however, is whether these correlative observations reflect a causal relationship, with inflammatory activation provoking ALS, or alternatively, ALS pathology triggering inflammatory responses.

A large amount of ALS research has made use of *in vitro* and *in vivo* models based on overexpression of genes with mutations linked to familial ALS. However, as 90% of ALS cases are sporadic, it is difficult to ascertain how representative these models are with respect to the human disease. This highlights one of the main obstacles currently limiting the study also of other neurological diseases: the need for patient-derived cell models that are relevant and robust enough to produce the large quantities of cells required for molecular and functional analyses. Current efforts on developing sALS models have used patient-derived samples from post-mortem brain, spinal cord and muscle 28,40. More recently, sALS patient-derived cell cultures have been developed including astrocytes differentiated from neural progenitor cells obtained from spinal cord necropsies 41, and induced pluripotent stem cells 42,43. All these models represent various degrees of compromise between relevance for understanding disease aetiology and suitability for diagnostics and drug discovery.

In this study, we have explored an alternative patient-derived neural model for the study of sALS disease: primary olfactory mucosa (OM) cultures derived from sporadic ALS patients. The OM is easily accessible for non-invasive biopsy in human adults 44. Either biopsied tissue or derived primary cultures have been shown to exhibit alterations in Rett's syndrome, Alzheimer's disease, fragile X syndrome, schizophrenia, Parkinson's disease and bipolar disorder with respect to healthy donors 45–50. OM-derived primary cultures contain several types of extraepithelial cells including multipotent stem cells 51–54 as well as olfactory ensheathing glia 55 which normally support the growth of primary olfactory axons from the neuroepithelium in the nasal cavity to the brain 56. These glia are present both in the peripheral as well as the central nervous system, and share characteristics of both astrocytes as well as Schwann cells. Moreover, OM-derived ensheathing glia have been used for therapeutic purposes in spinal cord injury 57,58 and even in ALS 59–61. The fact that OM transplants exhibit regenerative capacity in spinal cord 58, led us to speculate that the interaction of OM-derived cells with spinal cord-derived motor neurons may recapitulate the non-cell autonomous properties described for ALS.

It is conceivable that olfactory ensheathing cells (OECs) may share some similar characteristics of inflammatory cells, providing a degree of immunological protection against infections in the olfactory system. Indeed, microarray analysis has revealed that, relative to astrocytes and Schwann cells, OECs express higher levels of a number of innate immune factors, including lysozymes, chemokines and monocyte chemotactic proteins, suggestive of functions in modulating neuroinflammation 62. OECs have also been demonstrated to express TLRs and possess the cellular machinery to respond to certain bacterial ligands 63,64.

Another interesting aspect of using OM cells to model ALS is that it has been proposed that chemosensory impairment is an early symptom of many neurodegenerative diseases 65,66, including ALS 67,68. The mucus covering olfactory epithelium has been shown to contain reduced levels of growth factors that may be related to this reduction in olfactory capacity 68. Thus, this olfactory dysfunction suggests that mucosa neuroepithelium components might also be affected in neurodegenerative disorders, supporting their use as disease models. Notably, disease-specific alterations in gene expression, protein expression and cell function have previously been found in primary OM cultures derived from patients with schizophrenia and Parkinson's disease 69 as well as spastic paraplegia 70.

In this study, we propose the use of OM to model ALS, demonstrating their negative effect over motor neuron survival and morphology as well as the activation of inflammatory responses as a consequence of this deleterious interaction.

## Materials and methods

### Reagents and antibodies

All media such as Hank's balanced salt solution (HBSS), HBSS supplemented with Ca^2+^ and Mg^2+^, DMEM, DMEM:F12 and Neurobasal medium as well as other cell culture reagents including L-Glutamine, TrypLE, bovine pituitary extract, B-27 supplement, N-2 supplement were purchased from Gibco, Life Technologies (Barcelona, Spain).

Other special reagents used were: trypsin, penicillin, streptomycin, bovine serum albumin, trypan blue, retinoic acid and lipopolysaccharide (LPS), all purchased from Sigma-Aldrich (St. Louis, MO, USA); DNase-I (Roche, Mannheim, Germany); foetal calf serum (FCS, Hyclone, Logan, UT, USA); forskolin (Alomone, Jerusalem, Israel); primocin (InvivoGen, San Diego, CA, USA); carboxyfluoresceinsuccinimidyl ester (CFSE, Molecular Probes/Life Technologies, Eugene, OR, USA); brain-derived neurotrophic factor (BDNF, Santa Cruz Biotechnology, Santa Cruz, CA, USA), forskolin (Alomone Labs, Jerusalem, Israel); Smoothened agonist (SAG, Merck/Calbiochem, Darmstadt, Germany); Fluoromount-G (Southern Biotech, Birmingham, AL, USA); matrigel (BD Biosciences/Pharmingen, Madrid, Spain) and DAPI (Calbiochem, Nottingham, UK).

The antibodies against the following proteins were used: βIII-tubulin 71; S100β, SOD-1 and GAPDH (from Sigma-Aldrich); GFAP and nestin (from Chemicon, Temecula, CA, USA, now part of Merk-Millipore, Darmstadt, Germany); vimentin (Boehringer, Ingelheim, Germany); p75 and neuroligin (from Santa Cruz Biotechonology). The secondary antibodies used were anti-rabbit, antimouse and anti-goat Alexa 555, and Alexa 488 (Molecular Probes/Life Technologies) for immunofluorescence and peroxidase-labelled secondary antibodies (Sigma-Aldrich) for Western blot.

### Culture of patient-derived OM

Human OM primary cultures were obtained and cultured as previously described 72. OM primary cultures were derived from human nasal endoscopic biopsies that were carried out by otolaryngologists (E. Scola and J. Medina) at the Gregorio Marañón Hospital, Madrid. Written informed consent was obtained from all healthy or ALS patients, and the study was approved by the Gregorio Marañón Hospital ethics committee. Pre- and post-nasal biopsy olfaction was evaluated in ALS patients and controls, according to the Barcelona Smell Test-24 (BAST-24), previously validated for the Spanish population 73. No significant changes were found between the ALS patient group and controls, either before or after nasal biopsy. Briefly, tissue samples were kept at 4°C in HBSS until processing by disaggregation with 0.05% trypsin in HBSS for 20 min, inactivation with one volume of FCS, followed by a 5 min. treatment with 0.01% DNAse-1 in HBSS supplemented with Ca^2+^ and Mg^2+^, after which the cell pellet was finally resuspended in ME medium: DMEM:F12 (1:1), 10% FCS, 2 mM glutamine, 20 μg/ml bovine pituitary extract, 2 μM forskolin, 50 μg/ml primocin. Unless specified, OM cells were grown as adherent monolayer cultures in ME medium.

### Immunofluorescence assay

Immunofluorescence analysis was performed as previously described 68 using the primary and secondary antibodies detailed above and followed by 10 min. incubation with DAPI (1/500). Coverslips were mounted with Fluoromount-G according to the provider's guidelines. Representative images were taken with an Axiovert200 (Zeiss, Oberkochen, Germany) inverted microscope coupled to a CCD camera using Metavue 5.07 software (Universal Imaging, Bedford Hills, NY, USA).

### Neuronal survival assay

Preparation of frozen stocks of differentiated neurons from human foetal spinal cord neural precursors was as previously described 76. For co-cultures with patient-derived glia, OM samples were first seeded in six-well plates (10^5^ cells per well). After 24 hrs, differentiated neurons were thawed out and labelled with 25 μM CFSE in PBS containing 1% bovine serum albumin, for 15 min. at 37°C. After labelling, cells were diluted with 5 ml of neuronal medium [DMEM:F12 supplemented with B-27 (20 μl/ml), N-2 (10 μl/ml), streptomycin (100 U/ml) and penicillin (100 μg/ml)]. The cells were then centrifuged and resuspended in 3 ml of neuronal medium at 37°C. The percentage of live neurons was determined by trypan blue exclusion and the labelled cells were seeded in neuronal medium over OM monolayers at a neuron:OM ratio of 1:6. Culture medium was changed partially three times per week. After 2 weeks of co-culture cells were detached with TrypLE, washed with PBS and resuspended in 200 μl PBS for flow cytometry analysis. The total number of CFSE-positive cells was counted in 110 μl of each sample using a FACS CantoII flow cytometer (Becton Dickinson Biosciences, San Jose, CA, USA) and the results analysed using FlowJo software (TreeStar Inc., Ashland, OR, USA).

### Olfactory mucosa survival assay

Olfactory mucosa cells were seeded in six-well plates (10^5^ cells/well). After 24 hrs, ME culture medium was substituted for neuronal medium (DMEM:F12 supplemented with 20 μl/ml B-27, 10 μl/ml N-2, 100 U/ml streptomycin and 100 μg/ml penicillin) after which the medium was renewed partially three times per week. Twenty-three days after seeding, cells were collected and the total number of surviving cells was counted in a FACS CantoII flow cytometer (Becton Dickinson Biosciences) and the results analysed using FlowJo software (TreeStar Inc.).

### Neuronal morphology assay

Differentiation of human foetal spinal cord neural precursors along the motor neuron lineage was carried out by culturing them in Neurobasal medium supplemented with N-2 (5 μl/ml), BDNF (50 ng/ml), retinoic acid (100 nM), Forskolin (5 μM) and streptomycin/penicillin (100 U/ml and 100 μg/ml, respectively) during the first 4 days and Neurobasal medium similarly supplemented with N-2, BDNF, retinoic acid, streptomycin/penicillin, and SAG (100 nM) for up to 3 weeks.

A subconfluent culture of OM cells was grown on round coverslips in 24 multi-well plates and differentiated neurons were seeded on top (5 × 10^3^ neurons per well) in Neurobasal medium supplemented with N-2 (5 μl/ml) and streptomycin/penicillin (100 U/ml and 100 μg/ml, respectively). The medium was changed partially three times per week. After 2 weeks of co-culture, cells were fixed with paraformaldehyde and immunofluorescence analysis was performed as previously described 75 using 195 antiserum specific for βIII-tubulin 71 diluted 1:3000 during 1 hr followed by incubation with anti-rabbit Alexa 555 and DAPI. Representative images were taken and morphological quantification was performed by scoring a minimum of 15 βIII-tubulin-positive cells in at least nine random fields per condition into different morphologies:neuronal, when the cells exhibited filamentous βIII-tubulin staining and the length of the longest neurite was at least four times that of the longest axis of the nucleus; non-neuronal, when the βIII-tubulin stain was diffuse, cytoplasm was more expanded and its longer part was smaller than four times the size of longest axis of its corresponding nucleus. Cells not matching either morphology were discarded from the analysis.

### Lentivector production and titration

The viral vectors used to express wild-type and mutant SOD-1 were LentiSOD1^wt^ and LentiSOD1^G37R^ 18. The inflammation-responsive lentivectors LV-NFκBp-luc, LV-ESELECp-luc, LV-IL1-IL6p-luc encode the luciferase-IRES-GFP reporter construct under the control of 6× NFκB, E-selectin and IL1-IL6 hybrid promoters, respectively; the LV-SFFVp-luc lentivector encodes the same reporter under the control of the SFFV constitutive promoter 74. Lentiviral stocks were produced as previously described 75. Vector titre was determined in OM cells by infection with serial dilutions of the viral supernatants and the number of transduced cells determined 48 hrs post-infection by flow cytometry (FACSCalibur, BD Biosciences) using GFP expression.

### fALS cell model

The foetal human primary astrocyte cell line, HA1800, was obtained from ScienCell Research Laboratories (Carlsbad, CA, USA) and was cultured according to the provider's instructions. These cells were infected with LentiSOD1^wt^ and LentiSOD1^G37R^ 18 before each experiment.

### Innate immune response assay

Astrocytes and OM cells were infected with the indicated lentivectors 2 days after the treatment under study. For LPS stimulation, 24 hrs before treatment, cells were subjected to serum starvation by incubation overnight in DMEM containing 2% FCS to minimize interference by serum-borne factors. Cells were then cultured in the presence of LPS (500 ng/ml) in DMEM containing 2% FCS for the indicated period.

For co-culture, subconfluent spinal cord neural precursors were seeded in 96 multi-well plates coated with matrigel and differentiated along the motor neuron lineage by culture in Neurobasal medium supplemented with N-2 (5 μl/ml), BDNF (50 ng/ml), retinoic acid (100 nM), Forskolin (5 μM) and streptomycin/penicillin (100 U/ml and 100 μg/ml, respectively) during first 4 days and Neurobasal medium similarly supplemented with N-2, BDNF, retinoic acid, streptomycin/penicillin, and SAG (100 nM) during the last 5 days. Thereafter, OM cells and human astrocytes expressing SOD-1 (where indicated) and harbouring luciferase reporter constructs, were seeded on top of the neurons at a density of 7000 per well in Neurobasal medium supplemented with N-2 (5 μl/ml) and penicillin/streptomycin (100 U/ml and 100 μg/ml, respectively). Medium was changed every 2 days and luciferase assays were performed at the time indicated for each experiment.

### Luciferase assay

Cells were washed with PBS and frozen at −80°C. Luciferase activity was measured with an AutoLumat LB953 luminometer (Berthold Technologies, Bad Wildbad, Germany) using a commercially available assay system (E1501; Promega, Madison, WI, USA) following the manufacturer's instructions. All treatments were performed in triplicate. Normalized luciferase activity represented in the graphs was the result of dividing the relative light unit (RLU) values obtained for each inflammation-responsive vector by the mean RLU value obtained for the constitutive SFFV promoter vector under the same conditions.

### Western blot assay

Cells were collected, washed and resuspended in lysis buffer (50 mM Tris-HCl pH 7.5; 300 mM NaCl; 0.5% sodium dodecyl sulphate and 1% Triton X-100) and incubated for 15 min. at 95°C. Protein concentration of the extracts was measured using the Dc protein assay kit (Bio-Rad, Hercules, CA, USA) and 30 μg of each cell extract was resolved by electrophoresis in 12% polyacrylamide gels in the presence of sodium dodecyl sulphate. After electrophoresis, proteins were transferred to nitrocellulose membranes which were blocked with 10% low-fat milk in PBS-T (0.3% TWEEN 20 in PBS) and incubated overnight at 4°C with specific primary antibody against SOD-1 (1:1000) and anti-GAPDH antibody (1:5000). After washing, membranes were incubated with peroxidase-labelled secondary antibodies and the immunoreactive proteins were visualized using the enhanced chemiluminescence detection kit Western Lightning Plus-ECL (PerkinElmer, Waltham, MA, USA) following the supplier's instructions.

### Statistical analysis

Statistical comparison of the data sets was performed with Student's *t*-test. The differences are given with their corresponding *P*-value, which is the probability that the observed result could occur merely by chance under the null hypothesis.

## Results

### Co-culture of neurons with ALS mucosa cells diminishes their survival

**Figure 1:**
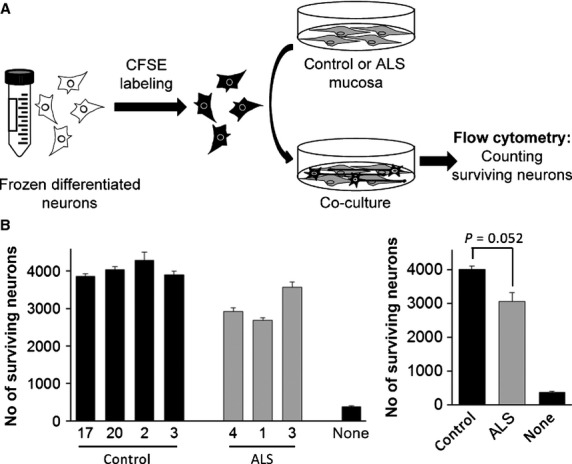
Spinal cord neuron survival after co-culture with olfactory mucosa (OM). (A) Scheme of the procedure using flow cytometry to study the survival of CFSE-labelled spinal cord neurons after co-culture with OM cells from healthy control or ALS donors. (B) Flow cytometry quantification of the number of CFSE-labelled surviving neurons after 2 weeks of co-culture with OM cells or in the absence of these cells (none). The left graph shows means and standard errors of the mean (SEM) of three independent assays of each OM sample. Samples are ordered by ascending patient age within each group (control and ALS). The right graph represents the combined mean and SEM of the control or ALS groups with the *P*-value for comparison of the means.

As a result of the fact that the majority of ALS cases are not associated with a known mutation 21, our first goal was to generate an ALS cell bank based on sporadic cases. Numerous studies have highlighted the relevance of glial cells in ALS pathogenesis 13–18 and therefore we concentrated on OM as it is a source of glia that can be obtained from living patients *via* a small nasal endoscopic biopsy 72. We generated a bank of OM from eight healthy donors and seven ALS patients (Table1). In this study, we used culture conditions optimized to enrich for the growth of glia cells 72; we successfully obtained primary cultures from all of the patients and did not detect any difference in the efficiency, survival, growth rates, life span or antigenic markers in samples derived from ALS patients compared to those from healthy donors (Figs S1 and S2, and Table1). All cells were similar in their expression of the antigenic markers GFAP, vimentin, S100β, neuroligin, nestin and the low affinity NGF receptor p75 (Fig. S2), all of which are characteristic of OECs. However, as a cautionary note, our previous work has demonstrated that these immunocytochemical properties do not clearly distinguish OECs from other cell types such as fibroblasts or gliomas 72. To test whether these biopsy-derived glia exhibit disease-specific neurotoxicity similar to that observed for spinal cord astrocytes derived from ALS patient cadavers, we co-cultured them with differentiated post-mitotic human spinal cord neurons 76 which we pre-labelled with CFSE to facilitate quantification. The neurons were plated over OM monolayers at a neuron:OM ratio of 1:6 (Fig.1A) and after 2 weeks of co-culture, cells were harvested and the total number of surviving neurons was counted by flow cytometry. We observed that neuronal survival was significantly higher when the neurons were co-cultured with OM derived from healthy donors compared to when they were co-cultured with OM from ALS patients (Fig.1B).

**Table 1:** Information about olfactory mucosa donors

Patient	Gender	Age	Maximum cell passage	Number of cells/mm^2^ for confluence
C1	Male	26	10	260
C2	Male	36	>10	364
C3	Female	51	>10	260
C16	Male	20	>10	260
C17	Female	25	>10	260
C18	Female	22	>10	260
C19	Male	29	>10	260
C20	Female	30	>10	364
ALS1	Male	57	>10	208
ALS3	Male	72	9	364
ALS4	Male	48	>10	208
ALS5	Male	36	10	208
ALS6	Female	61	>10	260
ALS7	Male	40	>10	364
ALS8	Male	39	>10	364

### Neuron morphology is aberrant in co-cultures with OM from ALS patients

**Figure 2:**
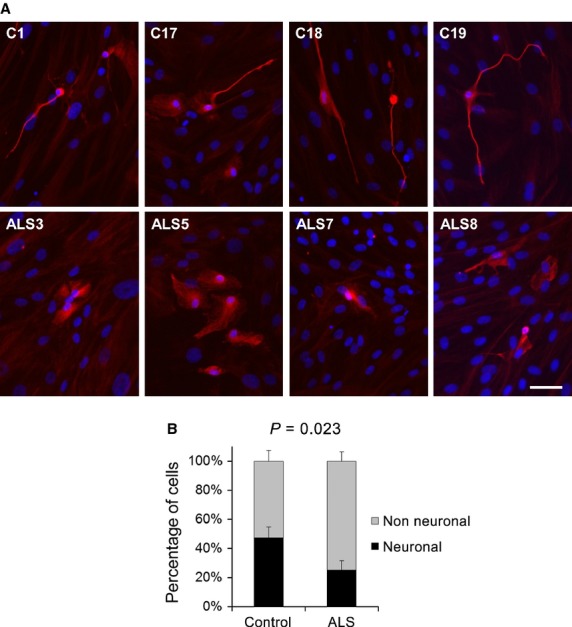
Morphology of the surviving spinal cord neurons after co-culture with olfactory mucosa (OM). (A) Representative immunofluorescence images of the surviving spinal cord neurons labelled for βIII-tubulin after 2 weeks of co-culture over monolayers of OM from four different healthy control donors (upper row) and four different ALS patients (lower row). Nuclei were labelled with DAPI. (B) Morphometric quantification of spinal cord neurons after co-culture over OM cells. CFSE-labelled cells were classified as: neuronal if they exhibited filamentous βIII-tubulin staining and the length of the longest neurite was at least four times the length of the longest axis of the nucleus; non-neuronal if they exhibited diffuse βIII-tubulin staining and all neurites were shorter than four times the length of the longest axis of the nucleus. A minimum of 15 neurons in at least nine random fields were scored for each OM sample. Graphs represent the percentage of cells exhibiting each morphology, showing means and standard errors of the mean of eight different controls and seven different ALS OM samples with the *P*-value for comparison of the means. The scale bar represents 50 μm.

In addition to diminished survival when spinal cord neurons were co-cultured with OM cells from ALS patients, we observed that the morphology of the surviving cells was significantly affected (Fig.2). While neurons grown over control OM cells preserved their typical morphology with a small cell body and long neuritis positive for strong fibrillar βIII-tubulin staining (Fig.2A, upper row), neurons grown over OM cells from ALS patients often displayed aberrant morphologies, exhibiting wider cell bodies and retracted neurites with less fibrillar and more diffuse βIII-tubulin staining (Fig.2A, lower row). As OM cells may express low levels βIII-tubulin 72, we optimized the βIII-tubulin staining sensitivity to exclusively label CFSE-positive cells. For morphometric analysis, cells were scored as neuronal if the length of the longest neurite was at least four times that of the longest axis of the nucleus. Based on this criterion, the data showed a significant reduction in the percentage of cells with neuronal morphology when the neurons were co-cultured with OM from ALS patients compared to when they were co-cultured with OM from healthy donors. We thus concluded that co-culture with OM derived from ALS patients not only reduced neuronal survival but also modified the morphology of surviving cells.

### Glial inflammatory response to LPS is not altered in ALS

**Figure 3:**
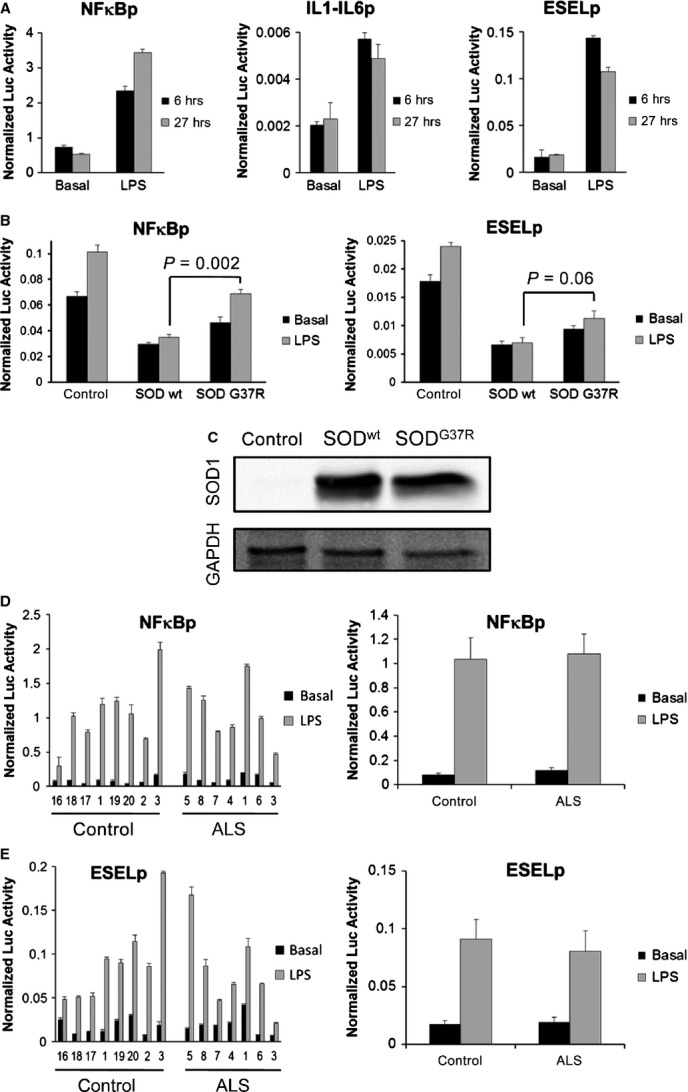
Study of the inflammatory response in ALS cell models. Cells were transduced with reporter lentivectors encoding the luciferase gene under the control of three inflammation-responsive promoters (NFκBp, an artificial promoter containing multiple NF-κB binding sites; IL1/IL6p, consisting of the human IL-6 promoter fused to the enhancer region of the human IL-1 promoter; or ESELp, the human E-selectin promoter) or the constitutive spleen focus-forming virus promoter (SFFVp). Normalized luciferase activity was obtained by dividing the luciferase activity measured in relative light units (RLU) for each inflammation-responsive promoter by the mean activity of the SFFVp reporter in the same conditions. Graphs represent means and standard errors of the mean (SEM) of three independent samples. (A) Reporter activities in untreated control olfactory mucosa (OM) cells (Basal) or after challenge with LPS (500 ng/ml) for either 6 or 27 hrs. (B) Reporter activities in control human astrocytes or those overexpressing SOD1^wt^ or SOD1^G37R^ either without (Basal) or with LPS challenge for 6 hrs. Comparison of mean luciferase activities (corresponding *P*-values are shown) after LPS treatment of astrocytes overexpressing SOD^G37R^ with those overexpressing SOD^wt^ revealed a significant increase using the NFκBp reporter; this effect was similar using the ESELp reporter, although not statistically significant. (C) Western blot analysis of SOD1 expression in the human astrocyte ALS cell models used in B. (D) The left graph shows luciferase activities of the NFκBp reporter in OM cells from eight control donors and seven ALS patients either without (Basal) or with LPS challenge for 6 hrs. Samples are ordered by ascending patient age within each group (control and ALS). The right graph represents the combined mean and SEM of the control or ALS groups. (E) Similar study to that shown in D but using the ESELp reporter.

Mounting evidence suggests that neuroinflammation plays an important role in the degeneration of motor neurons in ALS 77. It has been demonstrated that reactive astrocytes and microglia can release pro-inflammatory factors such as cytokines and chemokines, which are harmful to neighbouring neurons 78. To test whether OM cells from ALS patients have an altered pro-inflammatory state and/or a modified response to inflammatory stimuli, we transduced OM cells with three different inflammation-regulated lentivector systems 74. As a control, OM cells were transduced with the same reporter cassette (luciferase-IRES-GFP) under the control of a constitutively promoter (SFFVp). We first transduced OM cells from healthy donors and challenged them with a pro-inflammatory stimulus, LPS. We observed efficient activation of the inflammation-regulated promoters (NFκBp, IL1/IL6p and ESELp) after LPS treatment for 6 and 27 hrs (Fig.3A). As the largest responses to LPS were obtained with the NFκB and E-selectin promoters, we selected these reporters for subsequent experiments.

Our next goal was to ascertain if abnormal pro-inflammatory innate responses in ALS glia play a role in their toxicity to motor neurons. For this, we used the inflammation-inducible expression systems to first analyse human astrocytes overexpressing SOD1^G37R^, a previously published genetic model of ALS; in this model, the authors showed that co-culture with these modified astrocytes was deleterious to mouse motor neurons 18. We thus compared astrocytes expressing either wild-type SOD1 or the mutant SOD1^G37R^ to control astrocytes (non-transduced) after LPS treatment for 6 hrs (Fig.3B and C). Luciferase activities were lower in astrocytes than in OM cells, indicating less pro-inflammatory response in this cell type. In addition, NFκBp and ESELp reporter activity did not increase in astrocytes expressing either wt SOD1 or mutant SOD1^G37R^; overall luciferase activity was actually lower in transduced astrocytes compared to that observed in control astrocytes. In the case of NFκBp, reporter activity was significantly higher in the presence of mutant SOD1 than in the presence of wild-type SOD1. In addition, LPS challenge induced a significant increase in luciferase activity in presence of mutant SOD1, but not in the presence of wild-type SOD1 (Fig.3B). A similar effect was observed using the ESELp reporter although the differences were not statistically significant (Fig.3B).

We next used these inflammation-inducible reporter systems to study the inflammatory response in OM cells from healthy donors and ALS patients (Fig.3D). Although we observed efficient activation of both NFκBp (Fig.3D) and ESELp (Fig.3E) reporters in response to LPS treatment, no significant differences between ALS and healthy samples were observed, either in basal conditions or after LPS exposure. Thus, our results indicate that there is no alteration of the inflammatory response in OM cells from ALS samples.

### Olfactory mucosa from ALS patients shows increased inflammatory response in co-cultures with spinal cord neurons

**Figure 4:**
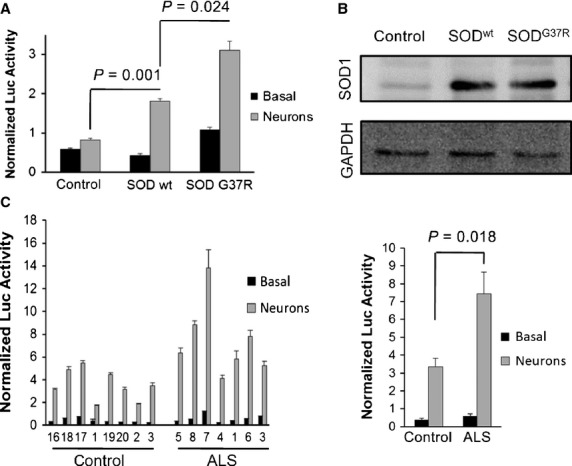
Study of the inflammatory response in ALS cell models co-cultured with spinal cord neurons. Cells were transduced with reporter lentivectors encoding the luciferase gene under the control of an artificial promoter containing multiple NF-κB binding sites (NFκBp) or the constitutive spleen focus-forming virus promoter (SFFVp). Normalized luciferase activity was obtained by dividing the luciferase activity measured in relative light units (RLU) for each inflammation-responsive promoter by the mean activity of the SFFVp reporter in the same conditions. Graphs represent means and standard errors of the mean (SEM) of three independent samples. (A) Reporter activities in control human astrocytes or those overexpressing SOD1^wt^ or SOD1^G37R^ cultured either alone (Basal) or with spinal cord neurons for 5 days (Neurons). (B) Western blot analysis of SOD1 expression in the human astrocyte ALS cell models used in A. (C) The left graph shows reporter activities in olfactory mucosa cells from eight control donors and seven ALS patients cultured either alone (Basal) or with spinal cord neurons for 1 day. Samples are ordered by ascending patient age within each group (control and ALS). The right graph represents the combined mean and SEM of the control or ALS groups with the *P*-value for comparison of the means.

We next posed the question if the altered inflammatory response described for ALS might be a consequence, rather than the cause, of the abnormal interaction between neurons and glia. To address this issue, we co-cultured human astrocytes, either unmodified (control), or overexpressing wild-type SOD1 or mutant SOD1^G37R^ with differentiated human spinal cord neurons (Fig.4A and B). As we had observed the largest response using the NFκBp reporter previously (Fig.3), we employed it to study the effects of only neuronal co-culture on the astrocytes (Fig.4A). Under basal conditions (in the absence of neurons), the SOD1-overexpressing astrocytes showed little change in reporter activity compared to unmodified astrocytes; neuronal co-culture significantly increased the luciferase activity in astrocytes overexpressing wild-type SOD1, and this increase was even greater in those overexpressing mutant SOD1^G37R^ (Fig.4A). Western blot analysis confirmed similar levels of expression of wild-type or mutant SOD1, ruling out the possibility that the different sensitivities to the neurons were because of different SOD1 levels. These results are consistent with the idea of the accumulation of wild-type SOD1 in sporadic ALS patients 79 to adopt an abnormal pathogenic conformation which may be exacerbated by overexpression or by certain point mutations.

We next applied the same methodology to measure the NFκB-mediated response in OM samples derived from healthy donors and ALS patients (Fig.4C). Again, a significant increase in reporter activity was observed when the cells were co-cultured with spinal cord neurons, with the OM cells from ALS patients showing higher luciferase activity than those from healthy donors (Fig.4C). As increased reporter activity in the ALS group is not observed in the absence of co-culture with neurons, we conclude that the altered glial inflammatory response in ALS is likely to be a consequence, rather than a cause, of neuronal death, which may liberate inflammatory cytokines which in turn activate NFκB-mediated responses.

## Discussion

Multiple lines of evidence have shown the immune system, including astrocytes and microglia, to be deleterious for motor neurons in ALS. Reactive astrocytes and microglia may release pro-inflammatory factors such as cytokines and chemokines which are harmful for the neighbouring cells 78. However, their role as the primary cause of the disease remains undetermined. Our data indicate that there is no increased innate immune response of glia in ALS: using the established model of SOD1 overexpression in human astrocytes, we did not observe increased pro-inflammatory response after LPS treatment and this result is similar when OM cells from healthy donors and those from ALS patients are compared. However, co-culture with motor neurons increases glial sensitivity to pro-inflammatory stimuli in ALS: we observed augmented NFκB-dependent reporter activity both in the SOD1-overexpressing astrocyte model as well as in OM cells from ALS patients. These results indicate that alterations in the innate immune response of glia in ALS might be a consequence of their interaction with damaged neurons rather than the cause of initial neuronal damage. Nevertheless, once sensitized, the modified pro-inflammatory response of glia in ALS could further worsen the state of neighbouring neurons. In agreement with this concept, previous work using a SOD1 transgenic mouse model demonstrated that microglia and T cells initially slow disease progression, but at later stages after accumulation of SOD1 protein, contribute to acceleration of the disease 80. Moreover, it has been shown in both ALS patients as well as in mouse models that activation of microglia and astrocytes takes place only after distal axon degeneration 40.

The use of OM to model ALS offers certain advantages over other cell models. The non-invasive and relatively simple nasal biopsy procedure provides a patient-derived source of living cells that can be easily expanded to perform molecular analysis and functional assays. Samples can be collected not only from patients showing genetic linkage but also from sporadic cases which represent the majority of ALS victims and are more difficult to model. Most importantly, in view that sporadic ALS patients may show wide variability in disease aetiology and responses to therapy, OM cells can be obtained from living patients, offering the future possibility of personalized *in vitro* drug screening prior to treatment of the patient. Presently, the majority of patient-derived sALS models originate from post-mortem tissue 28,40,41, placing limitations on the average cellular lifespan and not offering any benefit to the donor. The advent of induced pluripotent stem cells generated from sALS patients offers an expandable cell model that can be obtained from living patients 42, but this involves a complex, time-consuming and expensive protocol. On the contrary, OM cell culture is a relatively simple and reproducible technique which can yield long-lived cultures without the need for genetic manipulations which could generate undesired non-disease-related alterations. Furthermore, OM cultures avoid the necessity for inefficient, complex and expensive protocols to differentiate neural precursors as they provide a direct source of neural cells, which, as we have observed in the present study, recapitulate ALS-specific hallmarks. Firstly, we observed that compared to healthy donor OM cells, co-culture with those derived from ALS patients results in reduced survival and aberrant morphology of spinal cord neurons, in agreement with the deleterious effect of ALS glia on co-cultured neurons previously reported 14–18. This may be because of the generation of a toxic factor and/or decreased trophic support by the glia 26. The latter is particularly relevant in view of the fact that our controls show that neurons exhibit drastically reduced survival in the absence of mucosa cells (control samples ‘none’ in Fig.1B and 72). Secondly, we observed good correlation between the effects obtained with OM cells from ALS patients and those from a previously published ALS model using SOD1-expressing astrocytes 18. While LPS challenge did not result in increased NFκB-mediated response in ALS samples, when OM cells or astrocytes were co-cultured with neurons, an augmented sensitivity was observed in ALS samples that was not detected in the absence of neurons. These data indicate that while there may be no alteration of the innate immune response in cells from ALS patients, the differential death of neurons after co-culture with ALS-derived samples may trigger an inflammatory process including activation of NF-κB pathways. Consistent with this, NF-κB activation has previously been observed in spinal cord astrocytes in ALS patients as well as in TDP-43 animal models where it was also demonstrated that the inhibition of NF-κB with Withaferin A reduced denervation in neuromuscular junctions 81,82.

Although important advances in ALS research have been made using mutated SOD1-expressing astrocytes, this model has the disadvantage that it only represents a small fraction of genetic cases of the disease. Furthermore, transduction by the SOD1 transgene may result in overexpression the protein at non-physiological levels or generate other disease-unrelated artefacts such as insertional mutagenesis. In the present study, to achieve a completely human model, we have examined the effect of patient OM on motor neurons derived from human foetal spinal cord. In further work, it would be interesting to compare their effect on rodent motor neuron primary cultures, which can be prepared cells that are more fully committed to the motor neuron lineage 83,84. To facilitate such studies, the relative homogeneity of response to different ALS OM samples with respect to controls may permit the use of pooled patient cells or perhaps even immortalized cell lines to generate more user-friendly cell models for ALS. Validation of OM as a cell model for ALS offers a new versatile tool to accelerate research and therapeutic development for this presently incurable devastating disease. Detailed characterization of OM cell models opens up the possibility of correlating genetic and functional differences which will facilitate the identification of more cellular components implicated in the disease process.

## Supporting Information

**Figure S1** Survival of olfactory mucosa.

**Figure S2** Immunocytochemistry of OM cells cultured in glial medium.
