# Supplementary figures and images for: Patient-derived olfactory mucosa for study of the non-neuronal contribution to amyotrophic lateral sclerosis pathology
Source: J Cell Mol Med. 2015 Mar 25;19(6):1284–95. doi: 10.1111/jcmm.12488 (PMC4459844; doi:10.1111/jcmm.12488)

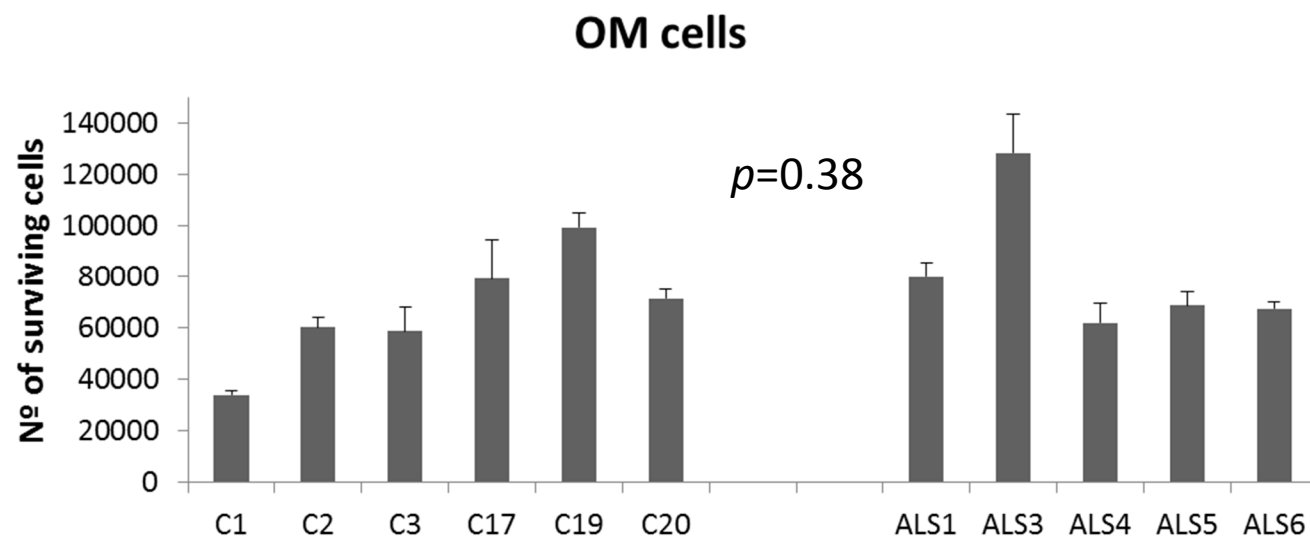

Survival of olfactory mucosa cells after 23 days in culture medium without growth factors

Supplement: Supplementary file 1 [file jcmm0019-1284-sd1.pdf]

## Controls

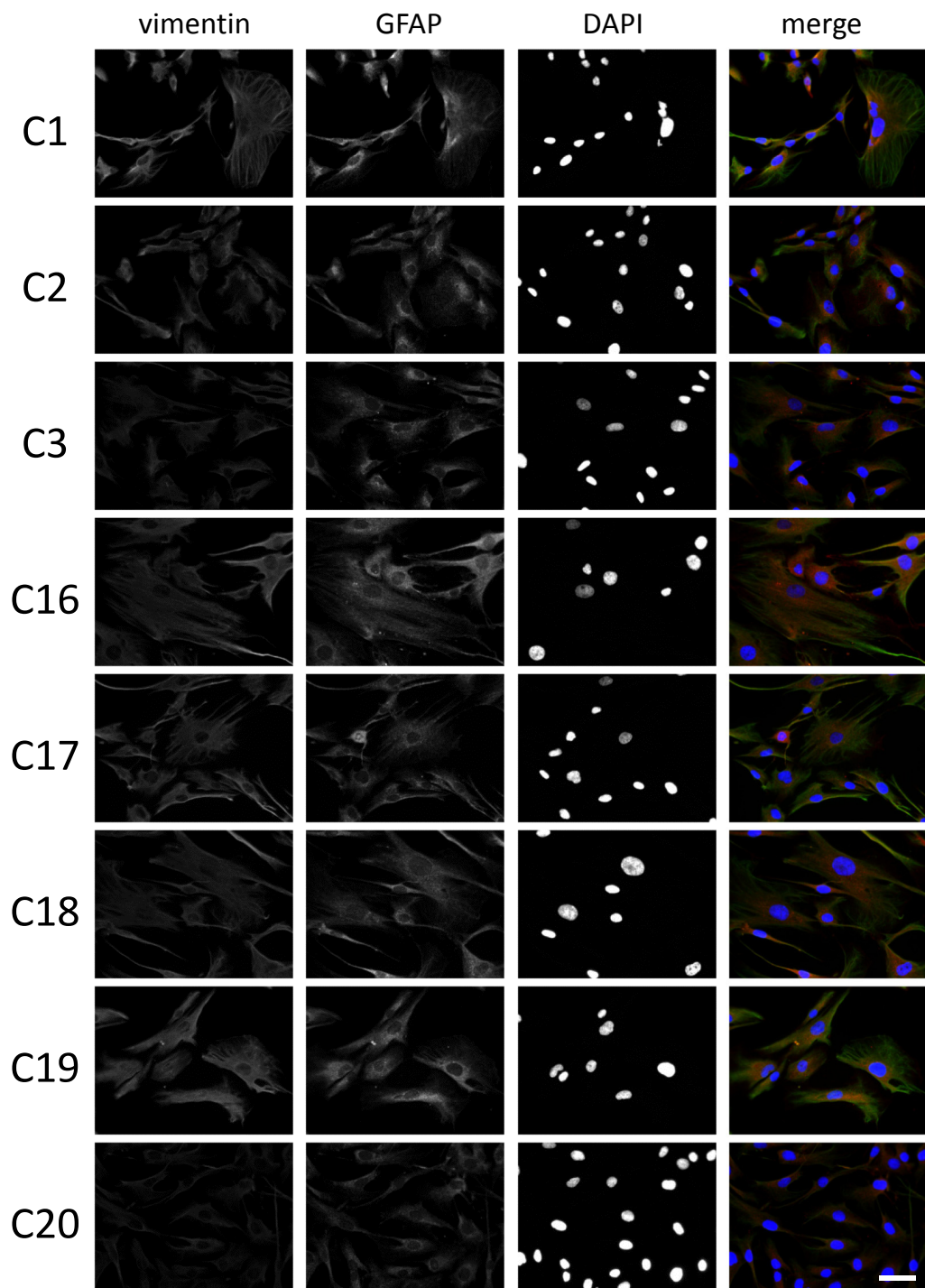

## ALS

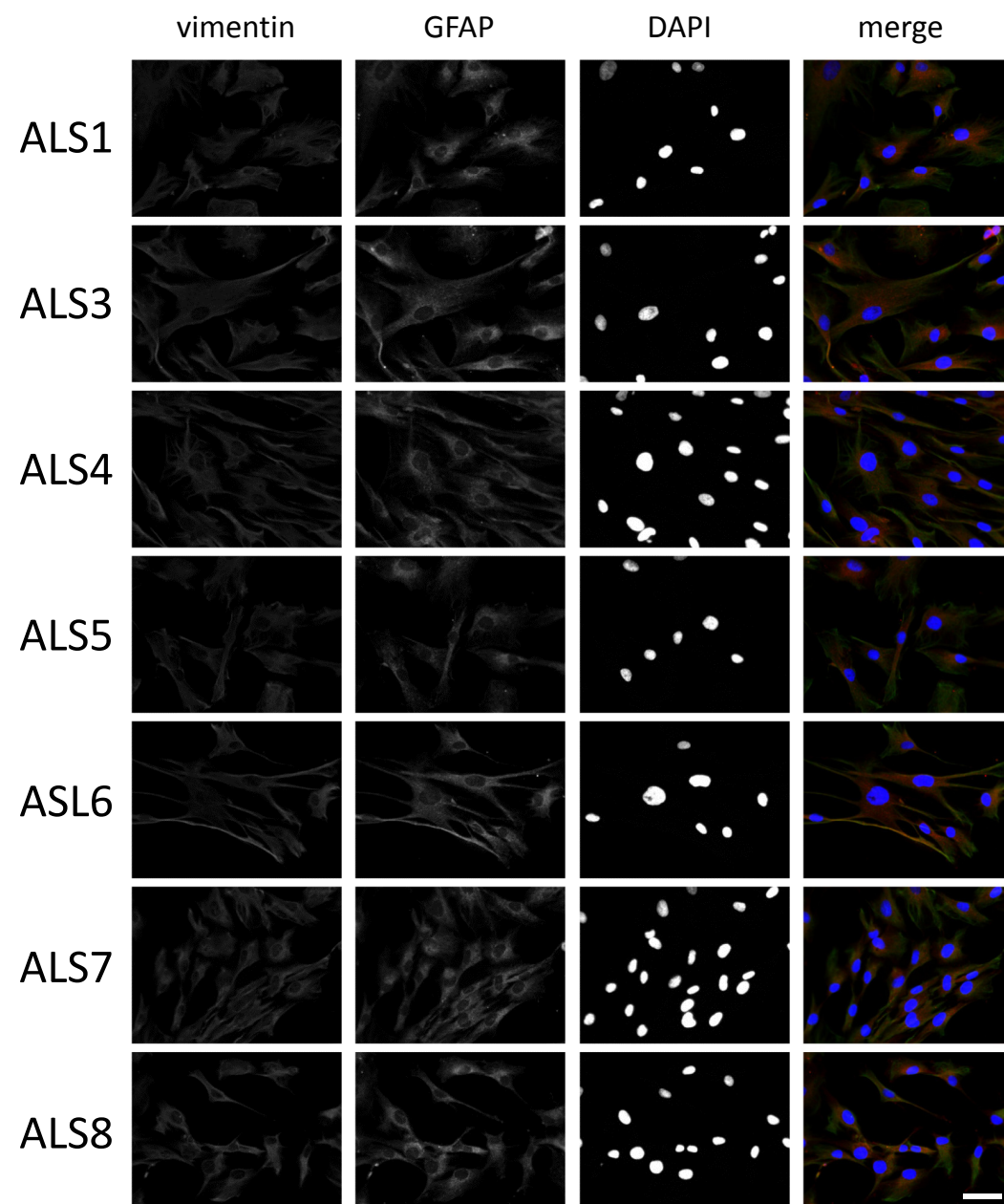

## Controls

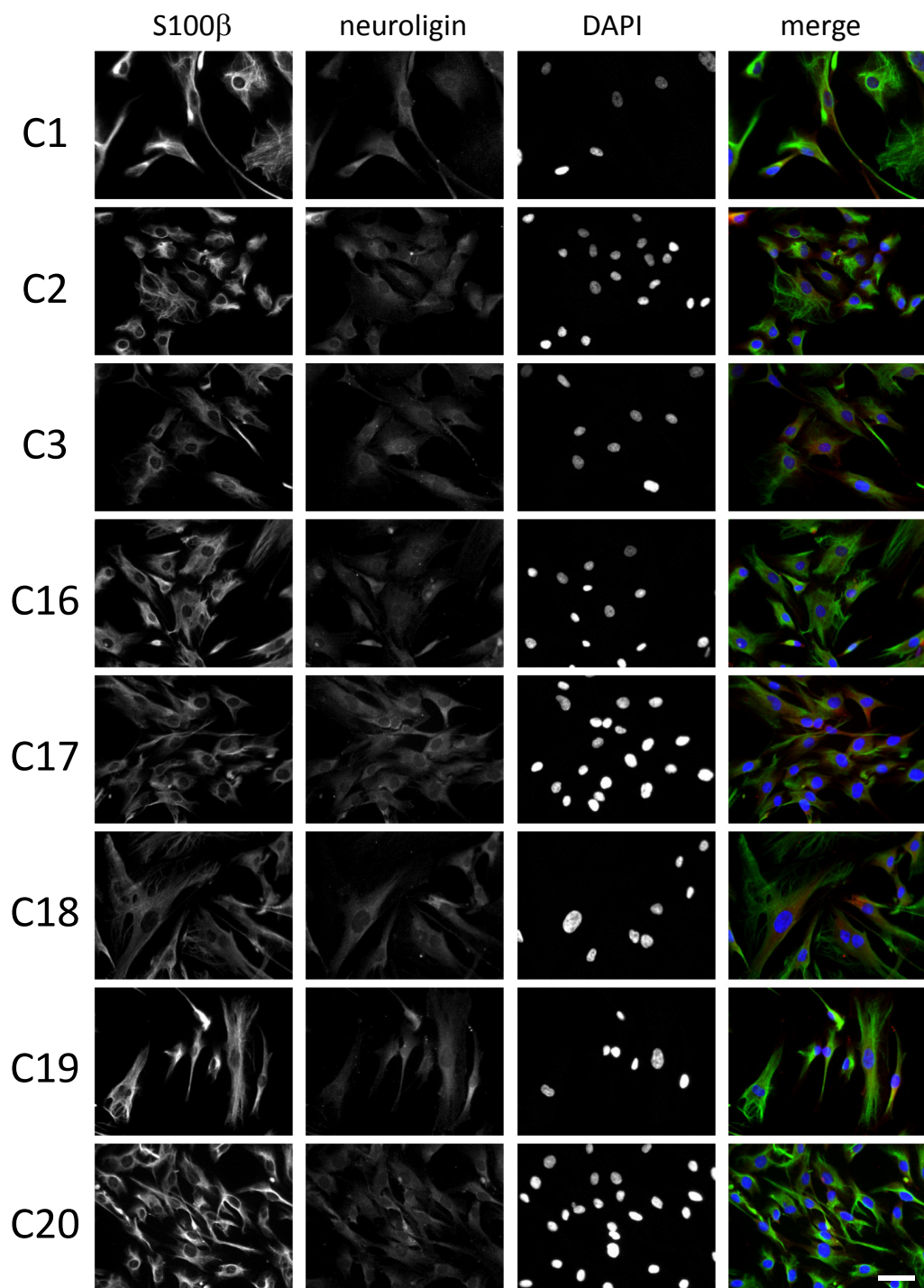

## ALS

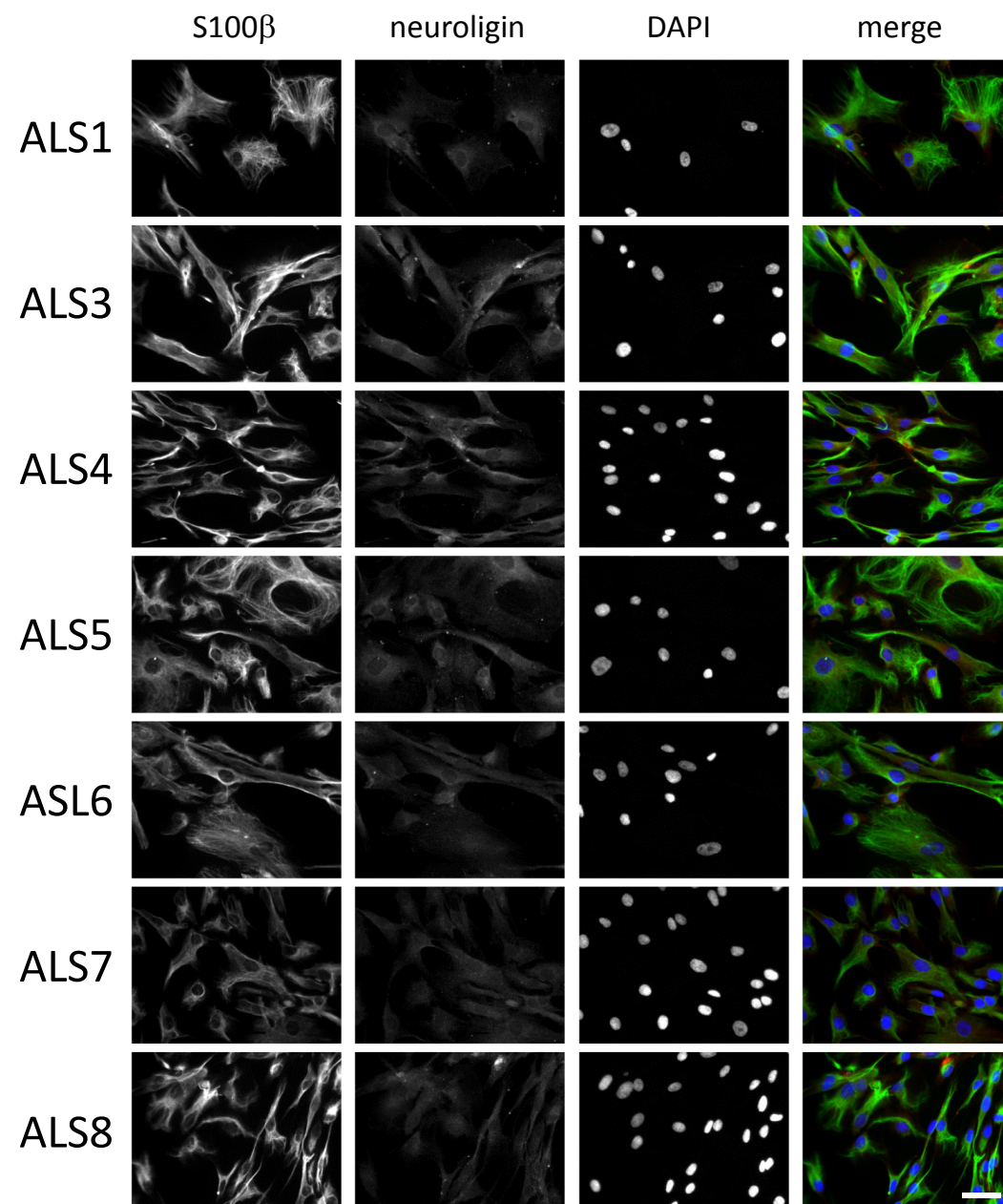

## Controls

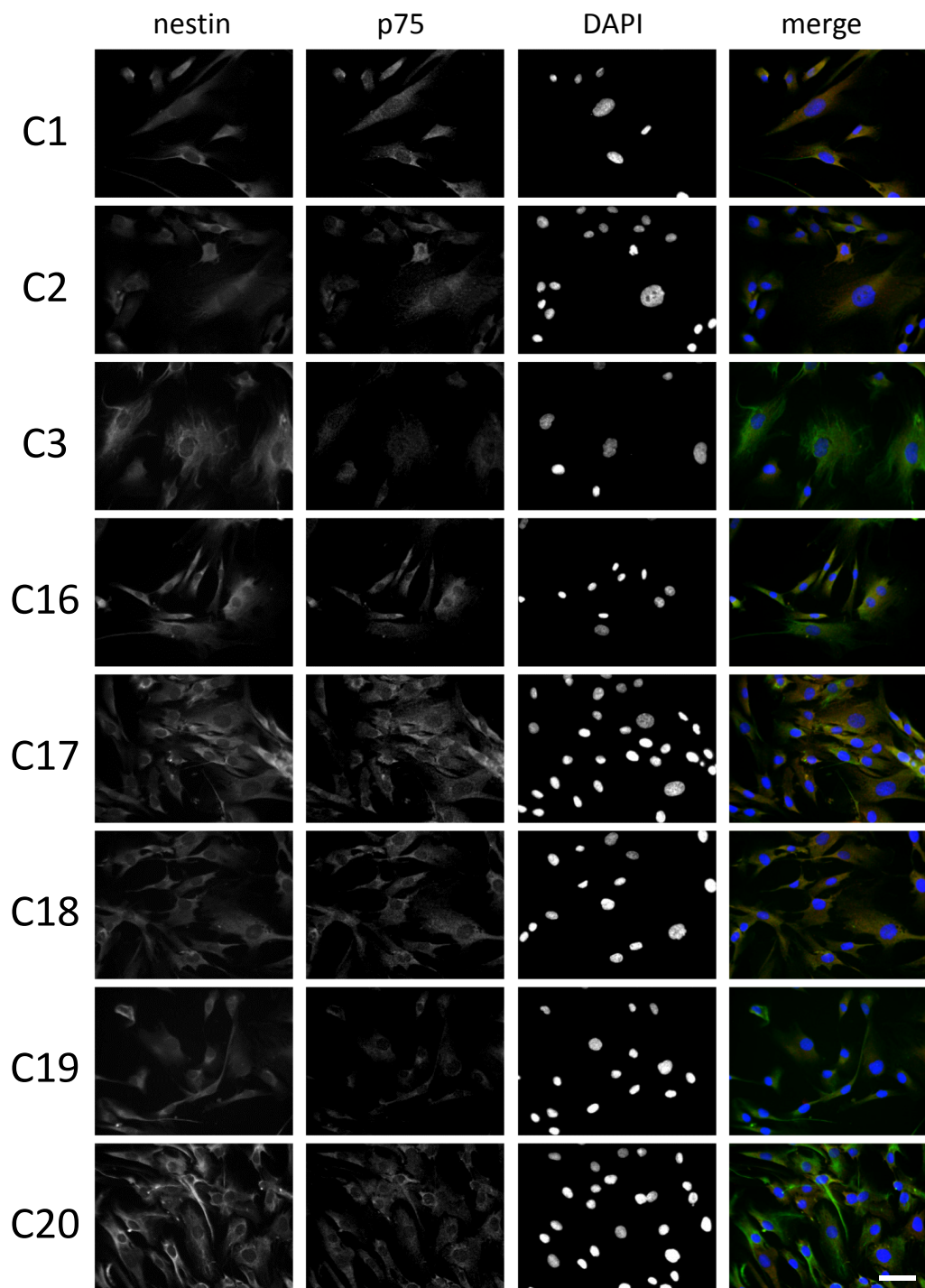

## ALS

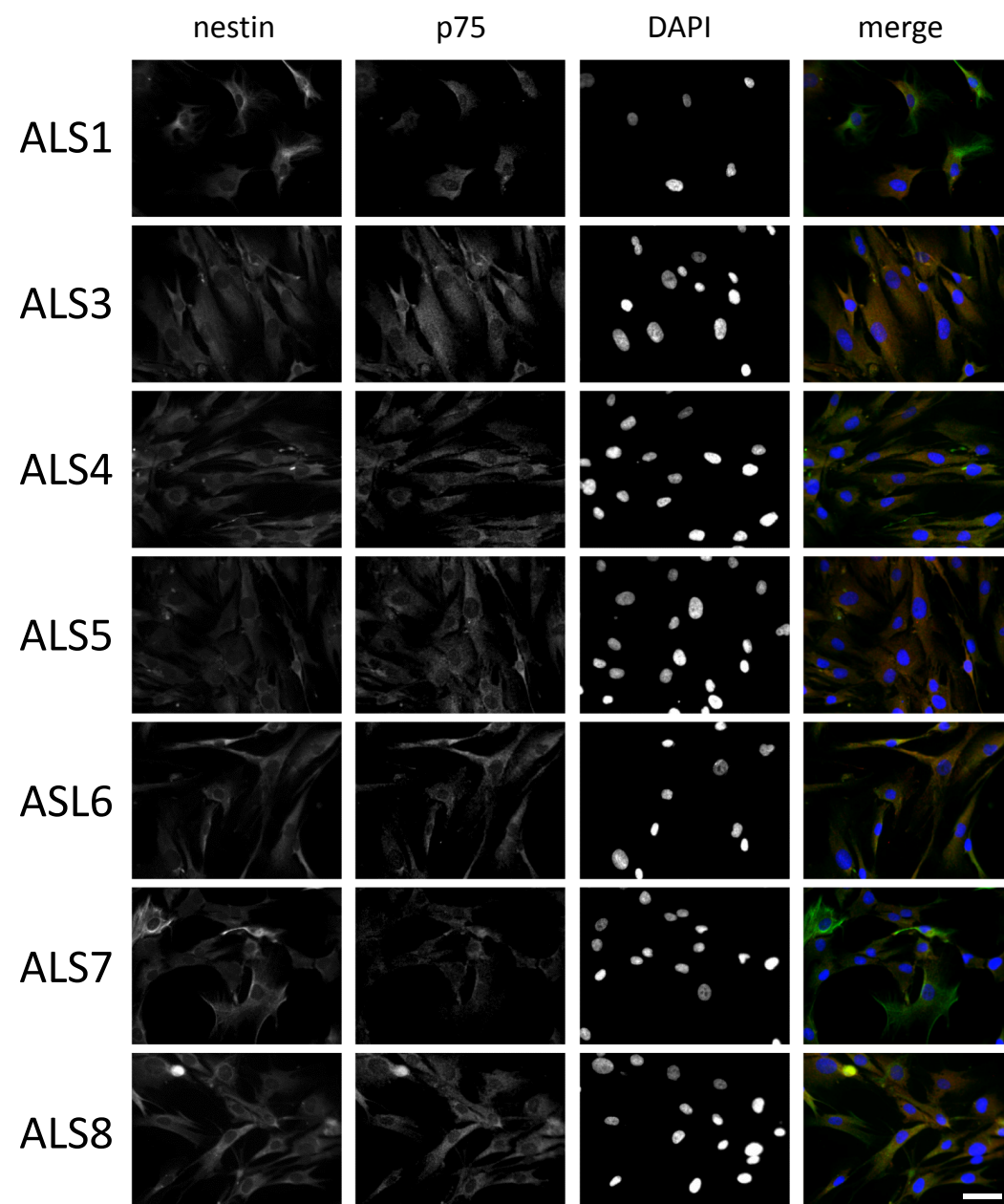

Supplement: Supplementary file 2 [file jcmm0019-1284-sd2.pdf]
